# Editorial: P2X7 Receptor Function in Neurodegenerative Diseases

**DOI:** 10.3389/fncel.2021.782014

**Published:** 2021-11-05

**Authors:** Robson Xavier Faria, Ricardo Augusto Melo Reis, Xavier Gallart-Palau

**Affiliations:** ^1^Oswaldo Cruz Institute, Oswaldo Cruz Foundation (Fiocruz), Rio de Janeiro, Brazil; ^2^Lab Neurochemistry, Biophysics Institute, Federal University of Rio de Janeiro, Rio de Janeiro, Brazil; ^3^Biomedical Research Institute of LLeida (IRBLLEIDA), School of Medicine UdL, Lleida, Spain

**Keywords:** purinergic receptors, neuron, ATP–adenosine triphosphate, inflammation, cell death, glia, cytokine

Purinergic P2 receptors are receptors expressed in the plasma membrane of several human cell types, underlying important physiological functions. The P2Y class encompasses eight subtypes of metabotropic receptors activated by different extracellular nucleotides. P2X class encompass seven subtypes of ionotropic receptors activated by ATP. The P2X7 subtype are trimeric ligand-gated cation channel receptors. Each unit presents three ATP-binding sites, impacting neuroinflammatory responses and regulation of the cellular function of neurons, microglia, and astrocytes. P2X7 receptors are activated by moderately high extracellular ATP concentrations (>100 μM) released from injured cells following brain injury or pathological conditions. P2X7 receptors are expressed preferentially on microglia and at a lower density on neuroglia (astrocytes, oligodendrocytes), participating in the release of proinflammatory cytokines. A series of articles updates the role of P2X7 receptor overexpression and its stimulation for microglia activation, leading to an elevated release of proinflammatory cytokines.

Territo and Zarrinmayeh reviewed the contribution, expression, and activation of P2X7 receptors in the CNS, on both neuronal and glial populations. The review focuses on the P2X7 receptor on microglia triggering the NLRP3 inflammasome, which induces the release of proinflammatory cytokines involved in chronic inflammation, pain, and cell death. Several factors are discussed as a consequence of the activation and proliferation of microglia activating TNFα, reactive oxygen species (ROS), NFκB signaling, and proinflammatory cytokines that impact neuronal death, glutamate-mediated excitotoxicity, and NLRP3 inflammasome activation.

Campagno and Mitchell reviewed the involvement of microglial cells in managing both immune response and debris clearance, as well as the P2X7 receptor central role in these functions. The authors show an increased expression of Nos2 and TNF alpha, and a decreased expression of Chil3 (YM1) in primary microglial cultures exposed to high ATP levels. Following lysosomal leakage, support for P2X7 receptor activation of the inflammasome emerged from data on cultured microglia showing that IL-1β release following receptor stimulation is inhibited by the cathepsin B blocker CA-074. Thus, the P2X7 receptor modulates the clearance of extracellular debris by microglial cells and mediates lysosomal damage that can activate NLRP3 inflammasome.

Dias et al. investigated the interplay between P2X7R and A2AR controlling neuroinflammation in rats subject to repeated restraint stress. The animals presented an anxious, depressive, and amnesic outline, together with increased expression of Iba-1, interleukin-1β and tumor necrosis factor α, and an up-regulation of P2X7 (mRNA) and A2A (receptor binding) receptors in the hippocampus and prefrontal cortex. Results demonstrate a functional interaction between P2X7 and A2A receptors controlling microglia reactivity likely involved in adaptive behavioral responses to stress. It also illustrates the cooperation between the two arms of the purinergic system in the control of brain functioning.

Oliveira-Giacomelli et al. reviewed the role of P2X7 receptors in immune responses during neurodegeneration. Inflammatory conditions, whether occurring peripherally or in the CNS, increase blood-brain barrier (BBB) permeability. P2X7 receptor may induce BBB disruption and chemotaxis of peripheral immune cells to the CNS, resulting in brain parenchyma infiltration. The authors reviewed how peripheral immune cells mediate the pathogenesis of Multiple Sclerosis and Parkinson's and Alzheimer's disease, mainly through T lymphocyte, neutrophil, and monocyte infiltration. They propose that P2X7 receptor activation contributes to progression of neurodegenerative disease beyond its known effects in the CNS.

Yin et al. show that palmatine, a protoberberine alkaloid used in Chinese traditional medicine, had inhibitory effects on P2X7 receptor expression in trigeminal ganglion and facial pain in trigeminal neuralgia rats. This severe and recurrent pain occurs in the distribution area of the trigeminal nerve, inflicting sufferings resembling knife cutting, acupuncture, electric shock, or burning. Results showed that palmatine administration could impair the mechanical pain threshold, significantly decrease the P2X7 receptor expression in trigeminal ganglion (TG), and lower IL-1β and TNF-α serum concentrations compared to the sham group. Additionally, p38 phosphorylation level in TG of trigeminal neuralgia (TN) rats was significantly decreased after treatment with palmatine. Thus, the data suggest that palmatine may alleviate mechanical facial pain in TN rats, possibly by reducing the expression of P2X7 receptor in TG, which may be attributable to inhibiting p38 phosphorylation and reducing IL-1β and TNF-α release.

Finally, Nurkhametova et al. show that nicotinic agonists can inhibit ATP-triggered release of the proinflammatory cytokines from immune cells via the so-called “cholinergic anti-inflammatory pathway.” This manuscript investigated the P2X7 receptor capacity for opening a pathway permeable to large molecules associated with activation of the inflammasome, and mouse peritoneal mast cells were treated with ATP alone or in the presence of acetylcholine and nicotine. Either acetylcholine or nicotine failed to inhibit the stimulatory effect of ATP on P2X7 receptors suggesting that cholinergic agents do not act on the initial steps of P2X7 receptor activation. It likely mediate the anti-inflammatory effect downstream of ATP-driven signaling, probably at the stage of the processing or releasing of the proinflammatory cytokines.

In conclusion, finding potent and selective P2X7 receptor inhibitors that are also CNS permeable and display acceptable pharmacokinetics has presented challenges for academic researchers and pharmaceutical companies. However, it is a consensus that the local concentration of ATP rises to levels close to P2X7 receptor stimulation upon neuroinflammation. This series reviewed novel mechanisms linking the proinflammatory cytokines released by P2X7 receptors with the inflammasome activation. Several P2X7 receptor antagonists have been studied in CNS disorders, and new preclinical trials have been conducted. Thus, the identification of the precise P2X7 receptor distribution on CNS combined with the development of high potency and selective modulators that are permeable to the BBB will represent possible progression in the field of P2X7 receptor study and for the treatment of CNS diseases.

## Author Contributions

All authors contributed to the article and approved the submitted version.

## Funding

This work was supported by CNPq (National Council of Research of Brazil) (RF holds a grant with Fellowship Process Number 308755/2018-9), FAPERJ (Research Support Foundation of the State of Rio de Janeiro) [JCNE (Young Scientist from Our State) with Fellowship process number E-26/203.246/2017] and Emergent Group of Research from Rio de Janeiro (E-26/211.025/2019). RR was supported by CNPq and FAPERJ [grant numbers E-26/202.668/2018, E-26/010.002215/2019, 426342/2018-6, and 312157/2016-9 and INCT-INNT (National Institute for Translational Neuroscience)].

## Conflict of Interest

The authors declare that the research was conducted in the absence of any commercial or financial relationships that could be construed as a potential conflict of interest.

## Publisher's Note

All claims expressed in this article are solely those of the authors and do not necessarily represent those of their affiliated organizations, or those of the publisher, the editors and the reviewers. Any product that may be evaluated in this article, or claim that may be made by its manufacturer, is not guaranteed or endorsed by the publisher.

